# EukRef: Phylogenetic curation of ribosomal RNA to enhance understanding of eukaryotic diversity and distribution

**DOI:** 10.1371/journal.pbio.2005849

**Published:** 2018-09-17

**Authors:** Javier del Campo, Martin Kolisko, Vittorio Boscaro, Luciana F. Santoferrara, Serafim Nenarokov, Ramon Massana, Laure Guillou, Alastair Simpson, Cedric Berney, Colomban de Vargas, Matthew W. Brown, Patrick J. Keeling, Laura Wegener Parfrey

**Affiliations:** 1 Department of Marine Biology and Oceanography, Institut de Ciències del Mar—CSIC, Barcelona, Catalonia, Spain; 2 Department of Botany and Biodiversity Research Centre, University of British Columbia, Vancouver, British Columbia, Canada; 3 Institute of Parasitology, Biology Centre, Czech Academy of Sciences, České Budějovice, Czech Republic; 4 Departments of Marine Sciences & Ecology and Evolutionary Biology, University of Connecticut, Storrs, United States of America; 5 Sorbonne Université, CNRS, Station Biologique de Roscoff, UMR7144, Roscoff, France; 6 Department of Biology, and Centre for Comparative Genomics and Evolutionary Bioinformatics, Dalhousie University, Halifax, Nova Scotia, Canada; 7 Department of Biological Sciences, Mississippi State University, Mississippi State, Mississippi, United States of America; 8 Department of Zoology, University of British Columbia, Vancouver, British Columbia, Canada

## Abstract

Environmental sequencing has greatly expanded our knowledge of micro-eukaryotic diversity and ecology by revealing previously unknown lineages and their distribution. However, the value of these data is critically dependent on the quality of the reference databases used to assign an identity to environmental sequences. Existing databases contain errors and struggle to keep pace with rapidly changing eukaryotic taxonomy, the influx of novel diversity, and computational challenges related to assembling the high-quality alignments and trees needed for accurate characterization of lineage diversity. EukRef (eukref.org) is an ongoing community-driven initiative that addresses these challenges by bringing together taxonomists with expertise spanning the eukaryotic tree of life and microbial ecologists, who use environmental sequence data to develop reliable reference databases across the diversity of microbial eukaryotes. EukRef organizes and facilitates rigorous mining and annotation of sequence data by providing protocols, guidelines, and tools. The EukRef pipeline and tools allow users interested in a particular group of microbial eukaryotes to retrieve all sequences belonging to that group from International Nucleotide Sequence Database Collaboration (INSDC) (GenBank, the European Nucleotide Archive [ENA], or the DNA DataBank of Japan [DDBJ]), to place those sequences in a phylogenetic tree, and to curate taxonomic and environmental information for the group. We provide guidelines to facilitate the process and to standardize taxonomic annotations. The final outputs of this process are (1) a reference tree and alignment, (2) a reference sequence database, including taxonomic and environmental information, and (3) a list of putative chimeras and other artifactual sequences. These products will be useful for the broad community as they become publicly available (at eukref.org) and are shared with existing reference databases.

## Introduction

Most lineages of eukaryotes (organisms with nucleated cells) are microbial, and eukaryotic diversity extends far beyond the familiar plants, fungi, and animals. Eukaryotic microbes—protists—include diverse lineages of mainly unicellular organisms that exhibit a wide range of trophic modes, life histories, and locomotion, including, for example, algae, heterotrophic flagellates, amoebae, ciliates, specialist parasites, and fungi-like organisms, among others. Although the term “protists” describes a polyphyletic assemblage, it was widely used for convenience to describe the smallest size fraction of eukaryotic organisms, delineating them from bacteria and archaea. Collectively, protists are important to ecological processes [1] and to human health [2]. Protists include important primary producers, particularly in aquatic ecosystems, as well as consumers that eat bacteria, algae, fungi, other protists, and even small metazoans, and thereby link microbial production to higher trophic levels. Other lineages of protists recycle nutrients as decomposers or live as symbionts of other organisms. In fact, animals (including humans) are routinely colonized by eukaryotic microbes that run the gamut from parasites to commensals to mutualists.

Environmental sequencing efforts over the last 15 years [3,4] have greatly expanded the known extent of eukaryotic diversity, and the pace of data generation continues to grow. These efforts have identified many apparently novel lineages that have never been cultivated, and have transformed our understanding of the environmental distribution of numerous taxa [5]. The majority of environmental sequence data is based on the small subunit ribosomal DNA (also called 18S rRNA) because it is universally present, has been sequenced for the most comprehensive array of known taxa, and has a combination of conserved regions for primer design and variable regions that enable taxon identification [6]. With the advent of high-throughput sequencing, millions of sequences from hundreds of microbial communities can now be rapidly characterized within a single study, enabling a broader community of researchers without a strong taxonomic background to investigate the temporal dynamics [7] and the spatial distribution of eukaryotic taxa within or across ecosystems [8–11], and from this, to test hypotheses about how eukaryotic communities are structured and how they respond to environmental change.

### Building a better database

**Fig 1:**
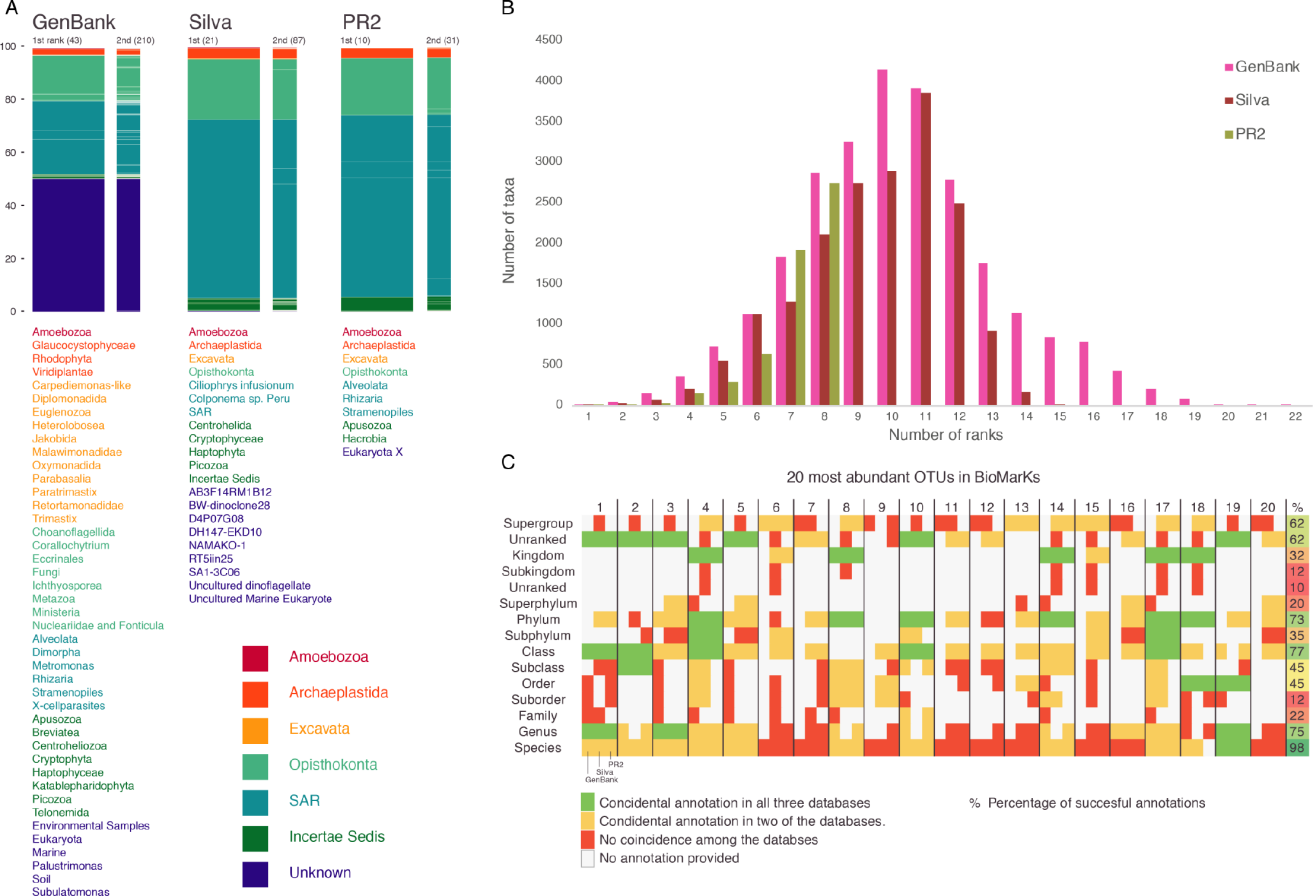
Comparison of existing databases. (A) Bar plots of taxonomic annotations of BioMarKs environmental sequences using the most popular reference databases for annotating 18S rRNA gene datasets for protist metabarcoding analyses (INSDC GenBank release 215, SILVA version 123.1, and PR^2^ version 4.2) at the first (level just below Eukarya) and second taxonomic ranks. White spaces in the boxes mark the changes between second-level ranks. Taxon names for the first rank in each database are listed below the bar plot. On top of each bar plot, within brackets, we show the number of taxa per rank. The taxon names for the first rank and bar plots are colored based on the eukaryotic supergroups defined by Burki 2014 [16]. (B) Distribution of the number ranks assigned to terminal taxa (unique taxonomic strings) in the three databases. (C) Taxonomic agreement on the annotation of the 20 most abundant OTUs within BioMarKs using each database, listed on x-axis as GenBank, SILVA, and PR^2^. Full taxonomic annotation available in S1 Table. INSDC, International Nucleotide Sequence Database Collaboration; OTU, operational taxonomic unit; PR^2^, Protist Ribosomal Reference Database; SAR, Stramenopiles, Alveolates, and Rhizaria; 18S rRNA, small subunit ribosomal DNA.

Environmental sequencing may be transformative in all the ways mentioned above, but the resulting datasets are only as good as the reference database used to annotate the data. Reference databases of ribosomal DNA bring together sequences from known isolates as well as Sanger-sequenced environmental datasets. The two main databases for eukaryotic ribosomal DNA sequences are SILVA [12], a general database that also includes Bacteria and Archaea ribosomal DNA, and the more specialized Protist Ribosomal Reference Database (PR^2^) [13]; many researchers also use the International Nucleotide Sequence Database Collaboration (INSDC), which encompasses the DNA DataBank of Japan (DDBJ), GenBank, and the European Nucleotide Archive (ENA) database resources [14]. These existing databases differ in numbers of sequences, taxonomic annotations, number of taxonomic ranks, and even inclusion of major lineages of eukaryotes [15] (Fig 1). Thus, the database used for annotation strongly influences the taxa and the taxonomic resolution reported in a given study.

While these databases have improved significantly in recent years, substantial challenges remain, largely because of changing views on eukaryotic relationships [16] and the influx of vast amounts of data from environmental sequencing that continues to reveal new lineages. This influx is reflected in the majority of sequences in INSDC that are annotated as unknown or environmental sequences from a given location (Fig 1A). These should ideally be incorporated into reference databases and novel clades assigned stable names so that they inform refinements in taxonomy of relevant eukaryotic clades. Stable names also ensure that new studies do not “rediscover” the same lineages, but rather refine what we already know about their diversity and distribution. Such efforts have greatly increased our knowledge of the diversity and ecological niche of novel lineages of marine stramenopiles [17], of *Blastocystis* within the human gut [18], and many more. A subtler challenge is how to handle the variable taxonomic ranks across clades of eukaryotes (Fig 1B). For example, vertebrates have 15 taxonomic ranks in the INSDC taxonomy, whereas the recently discovered Breviatea lineage has only three, despite the fact that it diverged prior to the split between animals and fungi [19]. This variability in ranks often reflects known diversity (there are only a handful of species known within Breviatea, compared with more than a million animal species) but nevertheless poses challenges during analysis. Many computational tools require a fixed number of ranks across taxa [20], and researchers generally want to be able to assess diversity across comparable clades with roughly equivalent divergence times. Ideally, databases should flexibly handle several taxonomic ranks in a way that enables researchers to use standardized levels when necessary.

To illustrate the impact of database choice on interpretation of a dataset, we annotated one dataset—BioMarKs, a survey of coastal marine microbial communities [9]—with the three major databases. At the highest taxonomic rank (the rank below “Eukarya”), this coastal marine survey appears to be dominated by unknown organisms identified as environmental sequences when annotated with GenBank, but to varying degrees by lineages in Stramenopiles (e.g., diatoms), Alveolates (e.g., dinoflagellates and ciliates), and Rhizaria (SAR) when annotated with SILVA and PR^2^ (Fig 1A). Going into greater detail, we compared the taxonomic annotation of the dominant taxa in BioMarKs using the three databases (Fig 1C, S1 Table). First of all, we had to normalize the annotation, because the ranks in the different databases do not represent the same taxonomic level. As expected, the different databases annotated a different number of ranks for each taxon, but we also found that the number of ranks between taxa was not the same for GenBank and Silva (PR^2^ has a defined number of ranks). Furthermore, for the taxonomic levels for which the annotation completeness was better, i.e., Genus and Species, the degree of agreement between databases was low; annotations agreed in only 15% at the Genus level and 5% at the Species level when comparing the three databases. The agreement between two of the annotations was 40% at the Genus level and 50% at the Species level. The situation was similar for the rest of the taxonomic ranks that showed a completeness higher than 50% (Class, Phylum, “Supergroup,” and the unranked level below Supergroup). The rest of the taxonomic levels were poor in terms of retrieved annotations and agreement between databases.

EukRef is a community effort funded by the Moore Foundation, the United States National Science Foundation, and the International Society of Protistologists. It aims to improve the taxonomic information associated to 18S rRNA sequences as well as assemble environmental metadata that provide context, and to create better reference databases for metabarcoding/amplicon studies. EukRef is part of the UniEuk project (unieuk.org), which aims to provide a comprehensive taxonomic framework for eukaryotes [21]. EukRef (Box 1) complements existing efforts curating multicellular eukaryotic taxa by filling in major gaps in our knowledge for the protists that comprise the breadth of the eukaryotic tree of life, and its products will be incorporated back into the established SILVA and PR^2^ databases. To be useful, database curation must be initially done by those with knowledge of the taxonomy, phylogeny, and ecology of a given group of interest so that informed decisions can be made during the curation process. Curators can incorporate relevant information from diverse sources such as traditional classification schemes, phylogenomic studies, historical literature, morphological observations, and distribution data from high-throughput sequencing studies [22]. Such expert knowledge enables researchers to generate robust classification schemes for lineages known only from sequences, such as the diverse marine stramenopiles (MASTs [23]), and can provide a mechanism for workers to link described organisms to proposed environmental MAST lineages (e.g., *Solenicola setigera* and *Incisomonas marina*, which belong to the MAST-3 [24,25] clade, or *Pseudophyllomitus vesiculosus*, which belongs to the MAST-6 clade [26]). Many parts of the eukaryotic tree of life are currently known either from sequences or from morphological records, but not both [27,28], so enabling morphological data to inform molecular classification is crucial [21].

**Box 1**

Definitions as used by EukRef:

- Low level: less inclusive taxon (e.g., genus),
- High level: more inclusive taxon (e.g., phylum),
- Database: refers to tab-delimited file constructed by EukRef curators containing information about the identity of a sequence, its classification, and environmental metadata,
- Clade: used here to refer to a clade in a phylogenetic tree,
- Taxon: a group of organisms that has been assigned a name in previous literature (e.g., a genus or a species),
- Group: a lineage or clade in a phylogenetic tree being curated,
- Chimera: DNA sequence that stems from two or more distinct sequences generated as a product of the DNA amplification process.

While ribosomal DNA is invaluable for taxonomic classification, this information alone is unable to reliably disentangle deep eukaryotic relationships and is most powerful when combined with insight from multigene analyses (including phylogenomics) and/or morphological data. To address this, EukRef supports a flexible curation approach that can incorporate expert knowledge as well as insight from multigene molecular analyses and morphological studies. This approach differs from that used by SILVA and Greengenes [29], which either rebuild the ribosomal RNA tree for the whole dataset from scratch (Greengenes) or insert sequences into an existing and fixed alignment and tree (SILVA). The use of backbone constraints based on published and robustly established relationships from phylogenomics and morphology facilitate incorporation of this knowledge and will be helpful in cases in which this is warranted by existing data. For example, Fungi is a very well-established group of eukaryotes but generally appears as polyphyletic in ribosomal DNA trees without backbone constraints [30]. EukRef guidelines recommend a tiered approach assessing multiple analyses to compare phylogenetic structure, with and without constraints, to understand their impact. In addition, we should be aware that novel lineages may have new insertions in ribosomal DNA or other differences that require rebuilding of the alignments. The improved reference database and phylogenetic tree will in turn enable better annotation of subsequent high-throughput sequencing studies.

### The EukRef curation process

EukRef is a platform where experts share the same guidelines and tools for the curation of taxonomic groups, with the fruits of these efforts to be reinvested into public databases. The initial phase of EukRef consists of development, coordination of experts, and yearly curation workshops and is done in partnership with UniEuk, a network coordinating a taxonomic framework for eukaryotes. Here, we present the standardized guidelines and open source operational tools developed by this initiative, also available through eukreg.org. The final outputs of EukRef for each group are (1) a phylogenetic reference tree and alignment, (2) a curated reference database with accession numbers, curated classification string, and curated metadata, and (3) a list of sequences known to be problematic (such as chimeras).

**Fig 2:**
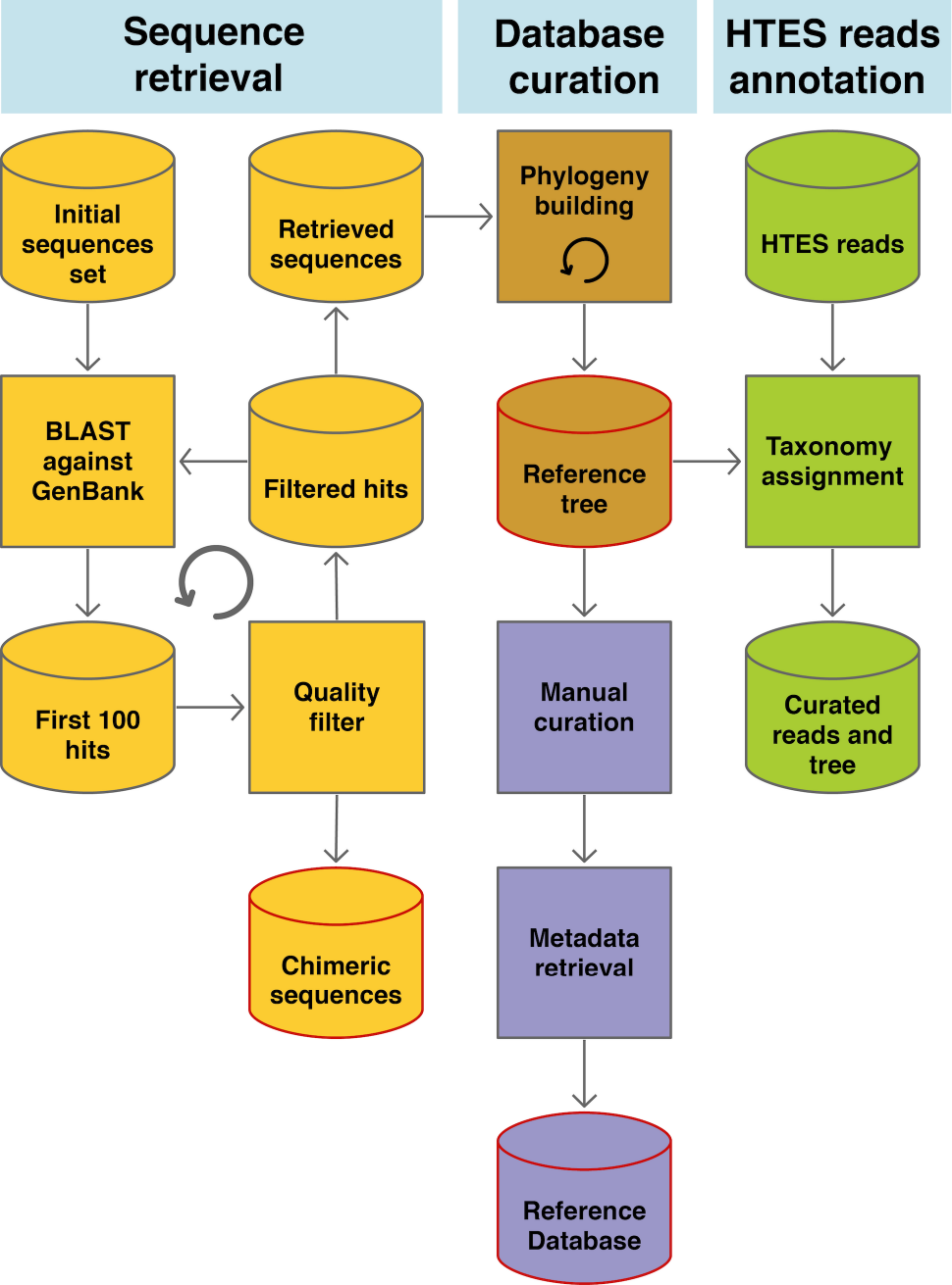
Simplified scheme of the EukRef workflow. Outputs are highlighted in red. HTES, high-throughput environmental sequencing.

To enable efficiency and consistency, the EukRef pipeline was developed to curate and annotate diverse eukaryotic lineages by researchers who are experts in that group, to comprehensively capture its existing sequence diversity (Fig 2). Curation starts with a broadly sampled alignment and corresponding 18S rRNA phylogeny. EukRef targets the18S rRNA gene because existing databases rely on this marker and because it best captures the breadth of eukaryotic diversity, but other markers could theoretically be used in a similar manner. This initial set of sequences is prepared by the curator and becomes the input to the EukRef workflow. The first step is an iterative retrieval of sequences from GenBank (INSDC) by BLAST [31] using a similarity threshold defined by the user, depending on the targeted lineage. During this step, sequences shorter than 500 bp and chimeras automatically detected by VSEARCH [32] are excluded. The expanded set of sequences retrieved from GenBank, together with the input sequences and relevant outgroups, are aligned using MAFFT [33] (a widely used and accurate alignment program [34], although curators can use another multiple sequence alignment program of their choice), then automatically trimmed using trimAl [35] and used for phylogenetic inference with RAxML [36], which can readily handle typical datasets of hundreds to several thousands of sequences. The resulting tree is the starting point for curation and classification of sequences. Curators then manually examine the tree to identify discrepancies such as long branches, which may be potential artifacts or chimeras that escaped the initial filtering, that should be removed (Fig 2). Following removal of these problematic data, a new alignment and tree are constructed with the remaining sequences. EukRef scripts then use the GenBank accession numbers for these sequences to retrieve the classification string and relevant metadata from GenBank and to organize this information in a tab-delimited file. This information, together with the tree, is the starting point for classification of the group and for each sequence. These outputs are combined with previous taxonomic knowledge, and improved metadata are manually incorporated throughout this process.

As a community, we have established guidelines for annotating sequences based on the phylogenetic tree and classification as part of the curation process, including guidelines for naming environmental clades. The curation process brings previous literature and expert knowledge to bear in annotating clades on the 18S tree generated in the EukRef pipeline, but current informal names are largely ad hoc, especially those referring to environmental sequence clades; some degree of consistency would be both simpler and more informative. The proposed annotation guidelines are designed to be practical, stable, and compatible with downstream analyses that will use the curated databases. We recommend a conservative approach that minimizes the introduction of new names by relying on published literature and only assigning names to well-supported clades. Summarized guidelines are listed below (Box 2). Detailed guidelines and examples can be found at eukref.org/classification-guidelines. The guidelines for consistent naming of novel environmental clades should prove particularly useful: attaching a name is a key first step forward in scientific communication, permitting the understanding of the extent of diversity and mapping the distribution of novel clades by allowing other scientists to recognize when they have found the same clade. Current ad hoc naming of these clades makes for substantial confusion when different arbitrary names are assigned to the same lineage, as inevitably happens.

**Box 2**

Classification guidelines

1. Clades should be supported by previous literature and/or receive statistical support in the 18S phylogenetic tree, if they are to be named.
2. Use names that are established in the literature. These can be formal taxon names, informal names, or environmental sequence clade names.
3. EukRef uses named rankless levels (i.e., not necessarily adhering to Linnaean classification ranks) following Adl and colleagues, 2012. Use as many levels as needed.
4. Only annotate to the level for which there is support. Fill in blank ranks by propagating down from the higher levels (more inclusive) to lower levels.
5. Do not name clades that are not supported or clades for which the applicability of a name is ambiguous (see website for examples and detailed guidelines).
6. Novel environmental clades may be named following the “Naming environmental clades” guidelines (below).

**Naming environmental clades**

- Only name lineages that are
  ○ well supported by bootstrap/posterior probabilities or possibly by clear 18S sequence signatures, and
  ○ composed of three or more clearly distinct sequence types, ideally from two or more different studies.
- Use a 3–5 capital letter code for the clade containing the environmental lineage. In most cases, this should be the most inclusive clade being annotated (e.g., “API” for environmental clades within Apicomplexa). Avoid using different codes for each subclade. This introduces unnecessary names and instability because the position of environmental lineages often shifts in subsequent analysis.
- Number the lineages in some arbitrary order, for instance, chronological order of their first appearance in a paper (e.g., API3). Use numbers again after an underscore for sublineages (e.g., API3_2).
- Never reuse the same number—even if a lineage later disappears—to avoid confusion (e.g., MAST-5 no longer exists).
- Do not name isolated sequences, especially long branches. These are potentially chimeras or low-quality sequences. When isolated sequences look genuine (are not chimeras, upon detailed inspection), they can be kept in the reference alignment and database because they may carry useful environmental information. These sequences should be identified simply by their clone name.

Our approach also provides tools for attaching biological and environmental information to each sequence in the curated database, including basic habitat information and whether a sequence came from a culture or morphologically identified isolate, or an environmental survey. Host associations are reported in the case of host-associated lineages (Box 3). To make this tool, we adopted standardized metadata annotation: Minimum Information about any (x) Sequence (MIxS) [37] and Environment Ontology (EnvO) [38]. The EukRef pipeline automatically assembles the complete set of sequences from National Center for Biotechnology Information (NCBI) that are associated with the clade of interest, but manual curation is required to vet the resulting phylogenetic tree, classify sequences, and transform the free text retrieved from GenBank into MIxS and EnvO standard inputs. Additionally, curators are encouraged to maximize the information attached to each reference sequence by using relevant literature to fill in missing metadata and flesh out fields retrieved from GenBank to maximize the information attached to each reference sequence.

**Box 3**

For the metadata annotation, we adopted standards from Minimum Information about any (x) Sequence (MIxS) and Environment Ontology (EnvO). Source is not included in MiXS but is required in EukRef.

- source*: Indicates if the sequence came from an isolate (culture or morphologically identified cell) or an environmental study.
- env_material: The environmental material entry refers to the material that was displaced by the sample or the material in which a sample was embedded, prior to the sampling event. Environmental material terms are generally mass nouns.
- env_biome: Biome defines the broad ecological context of a sample and is characterized by a certain biotic community and other environmental factors such as climate.
- biotic_relationship: Lifestyle, from free living to mutualistic symbiont.
- specific_host: For symbiotic lineages (including parasites). Host taxonomy ID (taxid) from INSDC.
- geo_loc_name: The geographical origin of the sample as defined by the country or sea name, followed by specific region name.

Altogether, these annotated datasets and the accompanying outputs are meant to provide a reliable tool for interpreting high-throughput sequencing surveys. The generated data will be available at the project website (eukref.org) and long-term hosted in GitHub (github.com/eukref/). Each lineage-specific dataset will be integrated into the UniEuk [21] (unieuk.org) taxonomic framework implemented at European Bioinformatics Institute (EBI) (www.ebi.ac.uk) and provide phylogenetic evidence for internal nodes and environmental clades of significance. EukRef will also provide reference trees to inform two other efforts within UniEuk, EukMap, and EukBank, which aim to improve eukaryotic taxonomic framework and to create a repository of properly annotated high-throughput environmental sequencing (HTES) reads, respectively [21]. In the long term, these datasets will also be transferred to existing reference databases for eukaryotes, including SILVA, PR^2^, and eventually INSDC. The annotations will also be freely available to other databases that are currently taxonomically restricted but might wish to expand to eukaryotes, such as the Ribosomal Database Project [39]. Ongoing curation and incorporation of newly available sequences will be facilitated by using Pumper [40], which allows an automatic sequence retrieval and tree building, and Sativa [41], which automatically annotates sequences in a tree. Both depend on the quality of the initial input, highlighting the need for high-quality initial annotation, as implemented in EukRef.

### Heterotrichea ciliates as a case study

**Fig 3:**
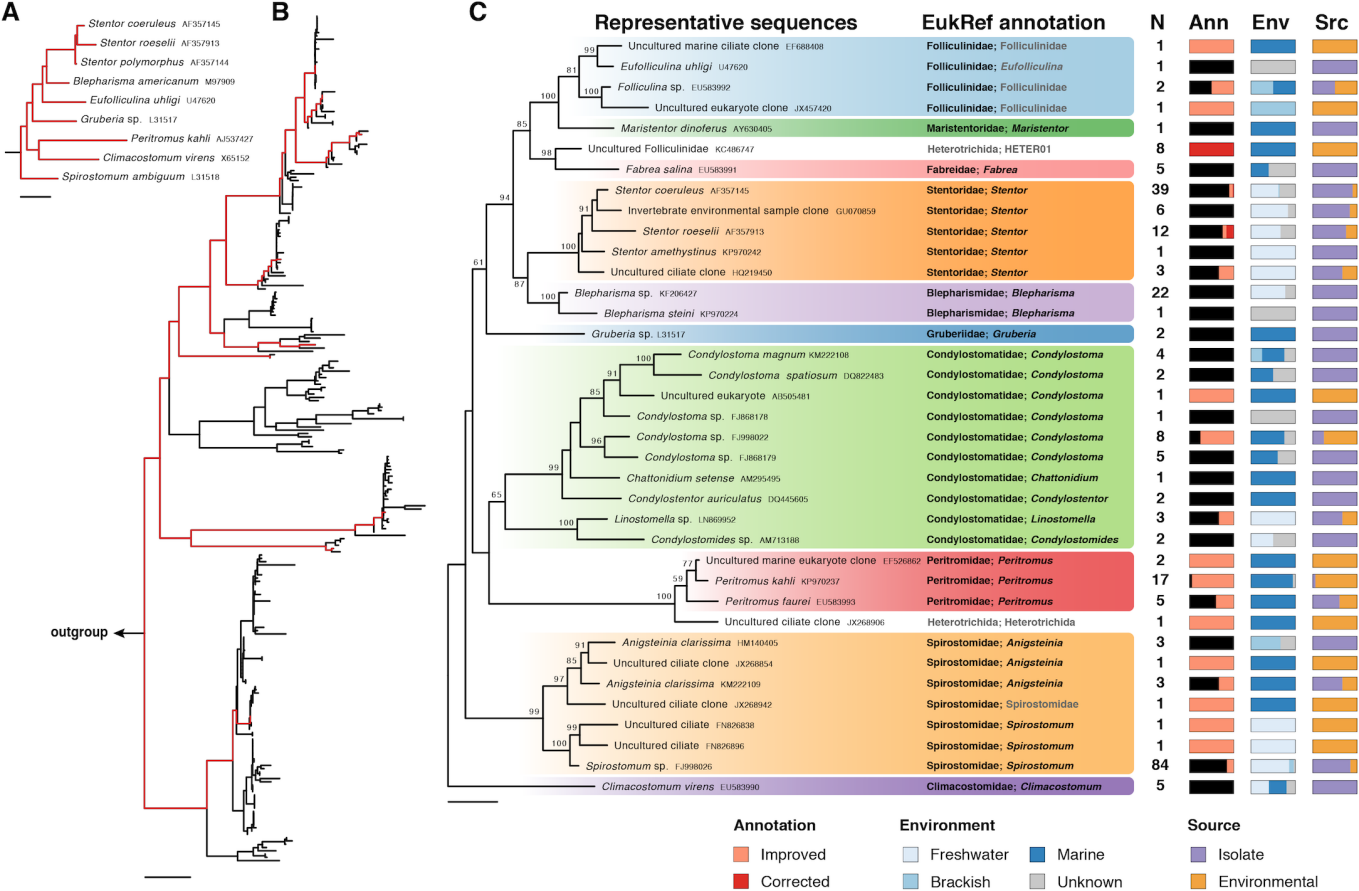
Case study of Heterotrichea, Ciliophora. (A) Phylogenetic tree of sequences used as input into the EukRef pipeline. (B) Phylogenetic tree following EukRef pipeline. Branches and leaves in red correspond to those present in the input dataset (in A); branches and leaves in black are those acquired by the EukRef pipeline and exclude artifacts and sequences discarded during curation that fell outside the group of interest. Output tree was used as a guide to perform the taxonomic annotation. (C) Output of EukRef curation. Representative sequences: output phylogenetic tree depicting representative sequences clustered at a 97% similarity threshold. EukRef annotation: taxonomic annotation following curation, which is propagated to all sequences in the 97% cluster (*N*, number of sequences in each cluster). Column Ann: proportion of sequences for which annotation was unchanged (black), improved (pink), or corrected (red). Metadata are added to the curated database for each sequence based on GenBank record and/or information in publications associated with the sequences. Column Env: portion of sequences in the cluster found in marine, freshwater, brackish, or unknown environment. Column Scr: portion of sequences derived from environmental sequencing or known isolates (either cultures or morphologically identified cells). Fully curated reference database of Heterotrichea available in S3 Data. Ann, annotation; Env, environment; Scr, source.

To illustrate both the curation process and why it is important, we annotated a well-known group of ciliates, the Heterotrichea, as a case study. The initial dataset imported into the pipeline consisted of only nine small subunit ribosomal RNA gene (SSU rRNA) sequences published by Rosati and colleagues in 2004 [42] (Fig 3A). After six cycles of the sequence retrieval script, we obtained 412 sequences, which were combined with outgroup sequences and used to build an initial tree (Fig 2). After discarding all the sequences that fall outside of the Heterotrichea, were of low quality, or were shorter than 500 bp, we were left with 258 heterotrich sequences (Fig 3B), representing 37 operational taxonomic units (OTUs) clustered at 97% (Fig 3C). Of these 258 sequences, 74% corresponded to isolates (previously cultured and/or isolated taxa) and 26% corresponded to sequences known only through environmental surveys. The classification of each of the 258 sequences was annotated based on the phylogeny, and the metadata were curated using GenBank records and associated literature, according to the EukRef guidelines (Fig 3C). The classification was improved or corrected compared to the initial GenBank record for 25% of the sequences, and metadata were added for the majority (70% of the sequences). This example shows the efficiency of the EukRef pipeline in collating both known isolate sequences and unidentified environmental sequences, thus increasing the taxon sampling, diversity coverage, and phylogenetic resolution of the targeted clade. Retrieval of environmental metadata provided valuable information about the ecology and environmental distribution of this group, and the overall curation effort generated a resource to more accurately annotate ciliate reads from high-throughput environmental surveys. The reference database, reference alignment, reference tree, and a list of discarded chimeras (see S1, S2, S3 and S4 Data) are publicly available at the EukRef website and GitHub as part of the Ciliophora database [20].

### Outreach and training

Yearly week-long intensive curation workshops organized in different parts of the world represent the core of the curation effort. These workshops bring together advisers (taxonomic experts) with curators—typically students and postdocs—who are actively investigating the taxonomy and diversity of a particular eukaryotic lineage in order to curate a reference database for a particular eukaryotic group that will further their research efforts. The participants acquire the expertise in using the provided workflow and tools to gather and curate a ribosomal DNA database, and they gain experience working in a UNIX-based command line environment. The process of curating the classification and metadata for their retrieved sequences requires participants to delve deeply into the literature for their lineage, improving their taxonomic knowledge as well. These early career scientists also form connections to the community of researchers studying protist classification and environmental distribution, allowing them to expand their network and establish collaborations beyond the context of EukRef. So far, we have organized two workshops in Vancouver, Canada, in 2015 and Barcelona, Spain, in 2016, and we have one more planned for 2018 in Roscoff, France. A total of 60 scientists were involved in the first two workshops; 40 of them were students in charge of curating a part of the tree of the eukaryotes. The first workshop in 2015 was focused on two well-known groups of eukaryotes, ciliates and excavates. The ciliate curation has been recently published [22] and is available at github (https://github.com/eukref/curation), while the excavates curation team is currently preparing their curated database for publication. The second workshop centered on two groups that are generally overlooked in molecular studies, Amoebozoa and Rhizaria [27,28], and the annotation is ongoing. Classification schemes informed by EukRef curation have also been incorporated in roughly 10 additional publications to date (see eukref.org/publications-citing-eukref).

### Conclusions and future perspectives

EukRef brings together members of the community with expertise in the taxonomy of different eukaryotic lineages to curate available ribosomal DNA sequences from cultured isolates and morphologically identified organisms, together with those from environmental surveys, all within a phylogenetic framework. In the long term, EukRef aims to assemble a curated reference database of 18S rRNA gene sequences covering all eukaryotes. Taxonomists have the greatest knowledge of eukaryotic groups but are rarely involved in curating sequence databases and seldom use existing environmental data. However, these are exactly the people needed to make sense of the vast diversity revealed in these studies. Bringing together taxonomists and microbial ecologists will provide better reference databases, which in turn will improve the automatic annotation of the numerous eukaryotic environmental sequencing surveys increasingly being conducted by the broader research community.

### Source code

All source code for the EukRef pipeline is available from https://github.com/eukref.

## Supporting information

S1 Table Full taxonomic annotation of the 20 dominant OTUs within BioMarKs for each database. Because not all the databases provide the same number of levels and not all the levels represent the same taxonomic rank, the annotations have been adjusted in order to make each level equivalent from a taxonomic perspective. Green cells represent coincidental annotation in the three databases, orange represents coincidental annotation in two of the three databases, red represents no coincident among databases, and gray represents absence of annotation. OTU, operational taxonomic unit.
(XLSX)

S1 Data Case study output 1, Heterotrichea alignment. (FAS)

S2 Data Case study output 2, Heterotrichea tree. (TRE)

S3 Data Case study output 3, Heterotrichea curated reference database. (TXT)

S4 Data Case study output 4, list of identified chimeras. (TXT)

## Abbreviations

DDBJ: DNA DataBank of Japan
EBI: European Bioinformatics Institute
ENA: European Nucleotide Archive
EnvO: Environment Ontology
HTES: high-throughput environmental sequencing
INSDC: International Nucleotide Sequence Database Collaboration
MAST: marine stramenopiles
MIxS: Minimum Information about any (x) Sequence
NCBI: National Center for Biotechnology Information
OTU: operational taxonomic unit
PR^2^: Protist Ribosomal Reference Database
SAR: Stramenopiles, Alveolates, and Rhizaria
SSU rRNA: small subunit ribosomal RNA gene
18S rRNA: eukaryotic small subunit ribosomal RNA gene
